# Cardiovascular health assessed by life’s essential 8 in Israeli adults aged ≥65 years and its association with long-term cancer incidence

**DOI:** 10.3389/fragi.2026.1825900

**Published:** 2026-06-17

**Authors:** Ahmad Marzooq, Samah Hayek, Yariv Gerber

**Affiliations:** Department of Epidemiology and Preventive Medicine, School of Public Health, Gray Faculty of Medical and Health Sciences, Tel Aviv University, Tel Aviv, Israel

**Keywords:** aging, cancer, cardio-oncology, cardiovascular health, cohort studies, epidemiology, life’s essential 8, older adults

## Abstract

**Background:**

Cardiovascular disease (CVD) and cancer, leading causes of morbidity and mortality in an aging global population, share risk factors and frequently coexist. Their relationship remains incompletely understood, particularly regarding the link between cardiovascular health (CVH) and cancer incidence among older adults.

**Objective:**

To examine the association between CVH, assessed using Life’s Essential 8 (LE8), a composite measure of health behaviors and cardiometabolic factors, and cancer incidence in a nationwide sample of older adults.

**Methods:**

A cohort of Israeli adults aged ≥65 from the 2005–2006 National Health and Nutrition Survey. Baseline health behaviors and CVD risk factors were used to categorize participants according to LE8. Cancer data were obtained from the National Cancer Registry through December 2019. Cause-specific Cox proportional hazards models estimated hazard ratios (HRs) for incident cancer, adjusting for sociodemographic variables.

**Results:**

Among cancer-free participants at baseline [n = 1543; mean age (SD), 75 (6) years; 53% women; 39% with pre-existing CVD], LE8 scores ranged from 7–97 [mean (SD), 56 (14)]. LE8 scores were positively associated with education and residential socioeconomic status and were lower among men and Arab participants. Over a median follow-up of 11.7 years, 345 participants (22%) were diagnosed with all-site cancer. LE8 was inversely associated with cancer incidence (adjusted HR = 0.88, 95% CI: 0.79–0.98 per 1 SD increase). The association was evident primarily among CVD-free participants (HR = 0.84, 95% CI: 0.74–0.97).

**Conclusion:**

Higher LE8 scores were associated with lower long-term cancer incidence in older adults. These findings extend the relevance of LE8 beyond CVD prevention and suggest that maintaining favorable CVH in later life may be associated with a lower risk of cancer.

## Introduction

With aging, the function of the body’s organs and systems declines, including the immune system, leading to diminished responses to pathogens and chronic low-grade inflammation. These changes, together with other age-related biological processes, contribute to the development of multiple chronic conditions, including cancer and cardiovascular disease (CVD) (Boudoulas et al., 2022). As life expectancy increases globally, the burden of these diseases is increasing disproportionately among older adults.

Cancer and CVD are the two leading causes of death worldwide (Koene et al., 2016; GBD 2023 Cancer Collaborators, 2025). A systematic analysis from the Global Burden of Disease Study 2023 indicated that there were 18.5 million incident cancer cases and 10.4 million cancer deaths, contributing to 271 million disability-adjusted life years (DALYs), reflecting years of life lost due to premature mortality and years lived with disability. The number of cancer-attributable deaths increased by 72.3% from 1990 to 2023 (GBD 2023 Cancer Collaborators, 2025). As of 2023, the number of incident CVD cases was 60.9 million (a 92.8% increase since 1990), and there were 19.2 million CVD deaths and 437 million CVD DALYs globally (Global Burden of Cardiovascular and Risks, 2025). The majority of these events occur in individuals aged ≥70 years (Qu et al., 2024).

According to the International Agency for Research on Cancer (IARC) in 2020, Israel’s cancer incidence rate (240.7 per 100,000) was higher than the global average (201 per 100,000). Cancer mortality rates, on the other hand, are lower than the global average (Ministry of Health I, 2023).

In 2022, the American Heart Association (AHA) introduced the Life’s Essential 8 (LE8) score, a tool designed to characterize ideal cardiovascular health (CVH), which encompasses both positive health behaviors and traditional modifiable risk factors. LE8 includes diet, physical activity, avoidance of nicotine, body weight, and levels of blood pressure, lipids, and glucose (Lloyd-Jones et al., 2022). LE8 expanded upon Life’s Simple 7 (LS7) with an updated scoring system and the addition of sleep health as the eighth component. In the general population, the LS7 and LE8 scores have demonstrated a strong ability to identify high-risk groups for numerous cardiovascular outcomes (Sun et al., 2023).

A growing body of experimental and clinical evidence reveals commonalities in the biological mechanisms underlying CVD and cancer (Boudoulas et al., 2022; de Boer et al., 2020). Studies have shown a bidirectional relationship whereby the occurrence of one may influence the development or progression of the other (Bell et al., 2023; Hasin et al., 2013; Hasin et al., 2016; Strongman et al., 2019). This association may be driven in part by the relationship of atherosclerosis with specific cancer subtypes, which persists after adjusting for classic risk factors (Boudoulas et al., 2022). These shared mechanisms may be particularly relevant in older adults, in whom cumulative exposure to cardiometabolic risk factors and age-related biological changes converge (Evans et al., 2025).

Recent studies examining cancer outcomes have used AHA metrics reflecting CVH. In the Multi-Ethnic Study of Atherosclerosis (mean age, 62 years), an optimal LS7 score was associated with a 20% lower risk of cancer compared with an inadequate score (Ogunmoroti et al., 2016). An additional study based on United Kingdom Biobank data reported similar findings. Each 10-point increase in the LE8 score was inversely associated with overall cancer incidence, corresponding to a 3% and 5% reduction in risk among men and women (median age ∼57 years), respectively, and to lower cancer mortality in both sexes (Yang et al., 2025). In the Moli-sani Study of over 21,000 adults (mean age, 55 years), ideal CVH assessed via LS7 was associated with a 29% lower risk of cancer hospitalization compared with poor CVH (onaccio et al., 2023). Importantly, these studies were conducted primarily in middle-aged populations, limiting their generalizability to older adults, who bear the highest burden of both CVD and cancer.

The LE8 score provides a direct measure of CVH, and its potential association with cancer risk is supported by evidence linking several of its individual components to reduced cancer risk. However, comprehensive, long-term studies on the association between the LE8 construct and cancer risk in the elderly population are lacking. This cohort study leveraged data from a nationwide sample of Israeli older adults (mean baseline age, 75 years) with up to 15 years of follow-up. By using a highly accurate national cancer registry and a comprehensive survey encompassing clinical, cognitive, lifestyle, and sociodemographic variables, the study effectively addresses potential confounding factors and biases. We aimed to evaluate the association of LE8 with first-ever cancer diagnosis in older adults. We hypothesized that higher LE8 scores would be associated with a lower risk of cancer occurrence.

## Methods

### Study design and setting

The cohort comprised participants from the first National Health and Nutrition Survey of the Elderly in Israel (“Mabat Zahav”), conducted between 2005 and 2006 by the Israel Center for Disease Control and the Nutrition Department of the Israeli Ministry of Health (Goshen et al., 2019). The survey population consisted of a random sample of Israeli citizens aged ≥65 years, drawn from the sampling framework of the two major health maintenance organizations in Israel (Clalit Health Services and Maccabi Health Services), which together cover approximately 86% of all elderly individuals in Israel. Because the percentage of older Arab adults was relatively low (6%), Arab participants were oversampled to ensure a sufficient sample size (Shafran et al., 2024a). The final sample included 1852 community-dwelling participants who had resided in Israel for at least 1 year. Data were collected through face-to-face interviews conducted at participants’ residences (private homes or retirement homes) using a structured questionnaire. Of the participants interviewed, 53 were excluded due to severe cognitive impairment (n = 46) or incomplete questionnaires (n = 7), leaving 1799 participants (with online baseline data available (Israel Center for Disease Control ICDC, 2012)). Individuals with a history of cancer at baseline (n = 235) and those missing key sociodemographic or clinical variables (n = 21) were excluded. The final analytic sample comprised 1543 participants. All aspects of the study were approved by the institutional ethics committees (Approval no. 354616-SMC).

### Assessment of cardiovascular health

CVH was assessed using the LE8 construct, based on AHA definitions of eight CVH metrics. As specified below and shown in Table 1, several LE8 components were adapted due to data limitations, resulting in a modified LE8 (mLE8). Each metric was scored on a scale from 0 to 100, and the mLE8 score was calculated as the average of all available component scores, as recommended by the AHA (Lloyd-Jones et al., 2022; Sun et al., 2023). Participants with ≥5 available mLE8 components were eligible for analysis, and the total mLE8 score was calculated as the average of the available component scores.

**TABLE 1:** Original Life’s Essential 8 (LE8) metrics and scoring compared with the modified LE8 (mLE8) used in the present study.

​	Original LE8 scoring		Modified LE8 scoring	
Metric	Categories	Scores	Categories	Scores
Diet	Percentile	Points	Percentile	Points
	95–100	100	95–100	100
	75–94	80	75–94	80
	50–74	50	50–74	50
	25–49	25	25–49	25
	1–24	0	1–24	0
Physical activity	Minutes/week	​	Minutes/week	​
	**≥**150	100	**≥**150	100
	120–149	90	120–149	90
	90–119	80	90–119	80
	60–89	60	60–89	60
	30–59	40	30–59	40
	1–29	20	1–29	20
	0	0	0	0
Nicotine exposure	Smoking status	​	Smoking status	​
	Never smoker	100	Never smoker	100
	Former smoker, quit **≥**5 y	75	Former smoker, quit **≥**5 y	75
	Former smoker, quit 1 -<5 y	50	Former smoker, quit 1 -<5 y	50
	Former smoker, quit **<**1 y or currently using inhaled NDS	25	Former smoker, quit **<**1 y	25
	Current smoker	0	Current smoker	0
Sleep health	Sleep (hours/night)	​	Sleep (hours/night)	​
	7–9	100	7–9	100
	9–10	90	9–10	90
	6–7	70	6–7	70
	5–6 or ≥10	40	5–6 or ≥10	40
	4–5	20	4–5	20
	<4	0	<4	0
BMI	BMI level (kg/m^2^)	​	BMI level (kg/m^2^)	​
	<25	100	<25	100
	25.0–29.9	70	25.0–29.9	70
	30.0–34.9	30	30.0–34.9	30
	35.0–39.9	15	35.0–39.9	15
	≥40.0	0	≥40.0	0
Blood lipids	Non-HDL cholesterol (mg/dL)	​	Dietary cholesterol intake (mg/day)	​
	<130	100	<160	100
	130–159	60	160–199	60
	160–189	40	200–239	40
	190–219	20	240–279	20
	≥220	0	>280	0
Blood glucose	Level	​	Diabetes status	​
	No history of diabetes and FBG <100 or HbA1c < 5.7	100	Non-diabetes	100
	No diabetes and FBG 100–125 or HbA1c 5.7–6.4 (prediabetes)	60		
	Diabetes with HbA1c < 7.0	40	Diabetes	0
	Diabetes with HbA1c 7.0–7.9	30		
	Diabetes with HbA1c 8.0–8.9	20		
	Diabetes with HbA1c 9.0–9.9	10		
	Diabetes with HbA1c ≥ 10	0		
Blood Pressure	SBP and DBP (mmHg)	​	SBP and DBP (mmHg)	​
	<120/<80	100	<120/<80	100
	120–129/<80	75	120–129/<80	75
	130–139 or 80–89	50	130–139 or 80–89	50
	140–159 or 90–99	25	140–159 or 90–99	25
	≥160 or ≥100	0	≥160 or ≥100	0

BMI, body mass index; DBP, diastolic blood pressure; FBG, fasting blood glucose; HbA1c, glycated hemoglobin; HDL, high-density lipoprotein; HEI, healthy eating index; NDS, nicotine delivery systems; SBP, systolic blood pressure.

Diet: Dietary intake was assessed via a 24 h recall. The AHA proposed the Healthy Eating Index (HEI) 2015 as a method for assessing dietary quality (Lloyd-Jones et al., 2022; Sun et al., 2023), which was applied in this study, as previously described in detail (Goshen et al., 2022; Goshen et al., 2023).

Physical activity: Physical activity was assessed using self-reported minutes of moderate or vigorous physical activity per week, collected via a questionnaire, as described in detail previously (Cohen et al., 2020a; Shaked et al., 2022).

Nicotine exposure: Information on nicotine exposure was self-reported via questionnaire (Goshen et al., 2019; Israel Center for Disease Control ICDC, 2012). The AHA definition includes combustible cigarette use, other inhaled nicotine delivery systems (e.g., vaping devices and e-cigarettes), and exposure to secondhand tobacco smoke (20 points should be subtracted for exposure to active indoor smoking, that is, living with a smoker); however, these data were not available in our dataset. Nicotine exposure has been categorized into five groups: current smokers, 3 categories of former smokers based on years since cessation, and never smokers.

Sleep health: A new metric introduced in the LE8 construct, based on self-reported average hours of sleep per night. This was assessed through a questionnaire in our study (Ashri et al., 2024).

Body mass index: Weight and height were measured objectively to calculate BMI (kg/m^2^) (Shafran et al., 2024b).

Blood lipids: The AHA recommends non-HDL cholesterol to score this component (Lloyd-Jones et al., 2022; Sun et al., 2023). Because blood lipid measurements were unavailable, we used daily dietary cholesterol intake (mg/day) estimated from a 24 h dietary recall as a proxy. This metric reflects short-term dietary exposure rather than circulating lipid concentrations and was applied due to data limitations (scored according to (D'Agostino RB et al., 2008)). In a sensitivity analysis, the lipid component was reclassified based on self-reported physician-diagnosed hypercholesterolemia and/or lipid-lowering therapy (0 points assigned if either was present, and 100 points if neither was present).

Blood glucose: The AHA recommends scoring this component via fasting blood glucose or HbA1c and subtracting 20 points for individuals receiving glucose-lowering pharmacotherapy (Lloyd-Jones et al., 2022; Sun et al., 2023). Because blood glucose measurements were not available in our study, we scored this component based on self-reported physician-diagnosed diabetes.

Blood pressure: Blood pressure was measured with a digital monitor according to the AHA’s recommended protocol. Blood pressure levels were classified according to systolic blood pressure (SBP) and diastolic blood pressure (DBP), and 20 points were subtracted for drug-treated levels (Israel Center for Disease Control ICDC, 2012).

### Cancer incidence ascertainment

Cohort members were linked to the Israel National Cancer Registry via their national identification numbers. The Registry records all incident cases of malignant neoplasms (excluding basal and squamous cell skin cancers), carcinoma *in situ* and high-grade intraepithelial neoplasia, and benign neoplasms of the brain and central nervous system. The registry covers the entire Israeli population (approximately 9.1 million as of 2019) and has an estimated ascertainment rate of 97% for solid tumors (Cohen et al., 2020a). Information on date of diagnosis and diagnostic codes, assigned according to the International Classification of Diseases for Oncology, Third Edition, was obtained for primary cancer only (i.e., excluding metastases). These data enabled the identification of both incidence (first ever) and prevalent (pre-baseline) all-site cancers (codes C00.0-C80.9). Individuals with a previous cancer diagnosis were excluded from the analysis. Participants who remained free of cancer by the end of follow-up were censored at the date of death or the last cancer update date (December 2019). Data on all-cause mortality (available through the end of follow-up) were obtained by linking the cohort to the nationwide cause-of-death database maintained by the Ministry of Health.

### Cardiovascular disease assessment

Pre-existing CVD was self-reported during the baseline interview. Participants were classified as having CVD if they answered “Yes” to the following question: “Has a doctor ever told you that you have myocardial infarction, heart failure, or another heart disease?” or if they reported having undergone coronary artery bypass grafting or percutaneous coronary intervention (Israel Center for Disease Control ICDC, 2012; Shaked et al., 2022).

### Additional covariates

Information on age, sex, ethnicity (Jewish or Arab), years of education, chronic health conditions, disabilities, mental health, cognitive function, and other characteristics was obtained at the baseline interview (Goshen et al., 2019; Cohen et al., 2020a). Details of the questionnaires used in the study are available on the Ministry of Health website (an English version is available) (Israel Center for Disease Control ICDC, 2012).

Multimorbidity was defined as the number of preexisting chronic conditions and categorized as none (0), moderate (1-2 conditions), or severe (≥3 conditions) (Cohen et al., 2020a; Shaked et al., 2022). Mental health was assessed using the 12-item General Health Questionnaire (GHQ), with scores ranging from 0 to 36, with lower scores indicating better psychological wellbeing (Goodchild and Duncan-Jones, 1985). Cognitive function was assessed using the Mini-Mental State Examination (MMSE), with scores ranging from 18 to 30 (participants with ≤17 points were excluded). Higher scores indicate better cognitive function (Folstein et al., 1975). Neighborhood socioeconomic status (nSES) was determined by the Central Bureau of Statistics based on participants’ residential location, according to the 2008 census. Scores ranged from 1 to 20, with lower values indicating greater SES deprivation (Kheifets et al., 2022). Functional status was assessed using Katz’s Activities of Daily Living (ADL) score (Katz et al., 1963). In this study, participants who had six or more limited ADL were classified as impaired.

### Statistical analysis

Continuous variables were summarized as mean ± standard deviation (SD) and compared using ANOVA or the Kruskal-Wallis test, according to their distribution. Categorical variables were reported as percentages and compared using chi-square tests. To assess correlations among mLE8 components, Spearman coefficients were computed, and the results were visualized using a heatmap. Person-time of follow-up for each participant was calculated from time zero (study entry) until the first occurrence of cancer diagnosis, death, or study termination (December 2019), whichever occurred first. Cancer incidence rates for the overall study and by sex and mLE8 tertiles were estimated per 1000 person-years.

Primary analyses were conducted using cause-specific Cox proportional hazards models, with time on study as the time scale (Cox, 1972). Death without a preceding cancer diagnosis was treated as a censoring event. Hazard ratios (HRs) and 95% confidence intervals (CIs) were estimated for all-site cancer incidence per 1 SD increase in the mLE8 score. Multivariable models adjusted for age, sex, ethnicity, pre-existing CVD, years of education, and nSES. Covariates were selected based on a ≥5% change in the mLE8 coefficient between the unadjusted and adjusted models (Cohen et al., 2017; Greenland, 2008; Maldonado and Greenland, 1993). The proportional hazards assumption was tested using Schoenfeld residuals. A significant violation was shown for nSES; therefore, an extended Cox model including an interaction term between nSES and time was fitted.

Potential nonlinearity in the association between mLE8 and cancer risk was evaluated using penalized smoothing splines with four knots within the adjusted cause-specific Cox model. To determine whether the association differed by prevalent CVD, stratified analyses were performed. Among participants without CVD, cumulative incidence functions (CIFs) were estimated across LE8 categories, with death treated as a competing event using the Fine–Gray subdistribution hazards model, with multivariable adjustment consistent with the primary models (Fine and Gray, 2012; Wolbers et al., 2009). This approach enabled estimation of covariate-adjusted cumulative incidence in the presence of competing mortality.

Sensitivity analyses were conducted excluding (1) the smoking component, to evaluate whether the association was independent of smoking, and (2) the lipid component, owing to data limitations. Site-specific cancer analyses were not performed due to limited statistical power. All analyses were conducted using SPSS version 26 and R version 4.5.2, with p-values ≤0.05 considered statistically significant.

## Results

A total of 1543 participants free of cancer (mean baseline age ± SD, 74.46 ± 6.17 years; 53% women) were included in the analysis. Participant characteristics by mLE8 tertiles are presented in Table 2. mLE8 scores ranged from 7 to 97 (mean ± SD, 56.31 ± 14.44). Higher mLE8 scores were positively associated with education and nSES, and inversely associated with male sex, Arab ethnicity, and pre-existing CVD. Mean age did not differ across mLE8 tertiles. Cognitive function and psychological health were significantly higher among participants in the highest vs. lowest mLE8 tertiles. Lower mLE8 scores were associated with functional impairment.

**TABLE 2:** Selected baseline characteristics of study participants by tertiles of modified Life’s Essential 8 (mLE8) score.

​	​	mLE8 Tertiles			​
Characteristic	Overall N = 1543	Lower N = 527	Middle N = 497	Upper N = 519	p-value
Age (y)	74.5 (6.2)	74.3 (5.9)	74.6 (6.3)	74.5 (6.3)	0.7
Male	718 (47%)	263 (50%)	237 (48%)	218 (42%)	0.031
mLE8 score	56 (14)	41 (8)	57 (4)	72 (7)	<0.001
CVD	607 (39%)	241 (46%)	200 (40%)	166 (32%)	<0.001
Multimorbidity No. Of diseases	​	​	​	​	<0.001
0	143 (9.3%)	22 (4.2%)	52 (10%)	69 (13%)	​
1–2	696 (45%)	178 (34%)	234 (47%)	284 (55%)	​
≥3	704 (46%)	327 (62%)	211 (42%)	166 (32%)	​
Arab	276 (18%)	135 (26%)	93 (19%)	48 (9%)	<0.001
Education (y)	10.4 (5.3)	9.1 (5.4)	10.4 (5.4)	11.8 (4.7)	<0.001
Neighborhood SES	10.4 (4.2)	9.7 (4.2)	10.3 (4.4)	11.3 (4.0)	<0.001
Psychological health (GHQ score)	6.4 (3.02)	6.7 (3.01)	6.4 (3.01)	6.2 (3.02)	0.012
MMSE score	26.9 (3.6)	26.3 (3.9)	26.9 (3.8)	27.6 (3.1)	<0.001
ADL functional impairment	320 (21%)	151 (29%)	98 (20%)	71 (14%)	<0.001

ADL, activities of daily living; CVD, cardiovascular disease; GHQ, general health questionnaire; mLE8, modified Life’s Essential 8; MMSE, Mini-Mental State Examination; SD, standard deviation; SES, socioeconomic status.

Data are presented as n (%) or mean (SD), as appropriate.

**FIGURE 1:**
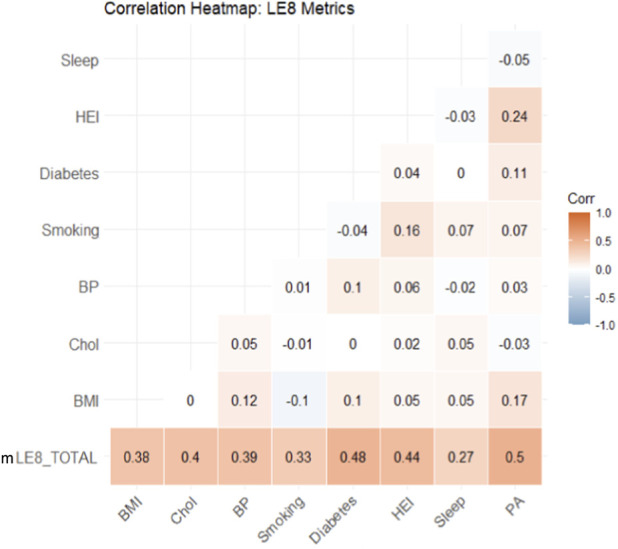
Heatmap of correlations between modified LE8 (mLE8) components. PA, Physical activity; mLE8, modified Life’s Essential 8; BMI, Body Mass Index; BP, Blood Pressure; Chol, Cholesterol.

The distribution of individual mLE8 components (Table 3) further characterizes the cohort’s cardiometabolic profile. Adverse CVH factors were common, including elevated blood pressure, obesity, diabetes, and low physical activity levels. In contrast, half of the participants reported optimal sleep duration, and over half were never smokers, whereas a smaller proportion achieved high diet quality. To assess the internal consistency of the mLE8 index, Spearman correlation coefficients were calculated between each mLE8 component score and the overall mLE8 score (Figure 1). All components were significantly correlated with the overall mLE8 score.

**TABLE 3:** Baseline distribution of modified Life’s Essential 8 (mLE8) components in the analytic cohort.

mLE8 component	n (%)
Diet quality, percentile	
Low HEI (<20)	390 (25.3)
Intermediate HEI (20–50)	774 (50.2)
High HEI (>50)	379 (24.6)
Sleep, hours/night	
<4	29 (2.2)
4–7 or ≥ 10	625 (47)
7–10	675 (50.8)
Blood pressure, mmHg	
SBP≥140/ DPB ≥90	796 (53.8)
SBP 130–139/ DBP 80–89	396 (26.8)
SBP<129 and DBP<80	287 (19.4)
Cholesterol dietary intake, mg/day	
>240	522 (34)
160–239	274 (17.9)
<160	738 (48.1)
Diabetes mellitus	
Diabetic	442 (28.7)
Non-diabetic	1100 (71.3)
BMI, kg/m^2^	
≥30	551 (37.3)
25–29.9	661 (44.7)
<25	267 (18.1)
Physical activity, min/week	
0–29	719 (46.6)
30–119	435 (28.2)
>120	389 (25.2)
Smoking	
Current smoker	183 (11.9)
Past smoker	503 (32.8)
Never	849 (55.3)

BMI, body mass index; DBP, diastolic blood pressure; HEI, healthy eating index; SBP, systolic blood pressure.

N varies across components due to missing data.

The median (25th-75th percentile) follow-up time was 11.7 (6.1–13.1) years. During this period, 345 participants (22.3%) were newly diagnosed with cancer. The most common cancer types [n (%)] were colorectal [47 (13.6%)], breast [41 (11.9%)], lung [39 (11.3%)], and lymphoma and leukemia [37 (10.7%)]. In addition, 548 participants died during follow-up without a preceding cancer diagnosis.

The overall cancer incidence rate, per 1000 person-years, was 23.4 (95% CI: 21.1–26.1); 27.8 (95% CI: 24.1–32.1) in males, and 19.9 (95% CI: 17.1–23.1) in females. Across the LE8 tertiles, the incidence rates were 25.2 (95% CI: 21.1–30.1), 24.2 (95% CI: 20.1–30.1), and 21.0 (95% CI: 17.4–25.2) for the low, moderate, and high mLE8 groups, respectively. The HRs for all cancers associated with mLE8 are presented in Table 4. Higher mLE8 scores were associated with lower cancer risk (adjusted HR = 0.88, 95% CI: 0.79–0.98, per 1 SD increase). This inverse association was more pronounced in CVD-free participants than in patients with pre-existing CVD. Further adjustment for multimorbidity, mental health, cognitive function, and functional impairment did not materially change the association (adjusted HR = 0.89, 95% CI: 0.80–1.00, per 1 SD increase). To reduce the likelihood of reverse causality, we excluded participants with cancer events occurring within the first 2 years of follow-up. The results remained consistent with the initial analysis (HR = 0.87, 95% CI: 0.77–0.98, per 1 SD increase).

**TABLE 4:** Hazard ratios (95% confidence intervals) for incident cancer associated with modified Life’s Essential 8 (mLE8).

Hazard Ratio (95% Confidence Interval)			
​	Cases/N	Unadjusted	Adjusted^*^
Entire cohort	345/1543	0.91 (0.82, 1.01)	0.88 (0.79, 0.98)
CVD-free	226/936	0.88 (0.77, 1.00)	0.84 (0.74, 0.97)
Pre-existing CVD	119/607	0.97 (0.80, 1.17)	0.99 (0.81, 1.21)
Excluding the smoking component^**^			
Entire cohort	343/1533	0.96 (0.86, 1.06)	0.92 (0.82, 1.03)
CVD-free	225/932	0.94 (0.83, 1.07)	0.90 (0.78, 1.03)
Pre-existing CVD	118/601	0.98 (0.81, 1.19)	0.99 (0.81, 1.21)
Excluding the cholesterol component^***^			
Entire cohort	341/1529	0.91 (0.82, 1.01)	0.85 (0.75, 0.95)
CVD-free	225/929	0.89 (0.78, 1.01)	0.82 (0.71, 0.94)
Pre-existing CVD	116/600	0.94 (0.78, 1.14)	0.93 (0.75, 1.14)
Complete data of all mLE8 components			
Entire cohort	272/1211	0.97 (0.86, 1.09)	0.93 (0.82, 1.05)
CVD-free	182/737	0.93 (0.81, 1.07)	0.89 (0.77, 1.04)
Pre-existing CVD	90/474	1.04 (0.84, 1.28)	1.02 (0.82, 1.28)

Estimates are per 1 standard deviation increase.

^*^ Adjusted for age, sex, ethnicity, pre-existing CVD, education, and neighborhood socioeconomic status.

^**^ Score based on 7 index components; multivariable model adjusted for age, sex, ethnicity, pre-existing CVD, education, neighborhood socioeconomic status, and smoking status.

^***^ Score based on 7 index components; multivariable model adjusted for age, sex, ethnicity, pre-existing CVD, education, neighborhood socioeconomic status, and cholesterol dietary intake status.

**FIGURE 2:**
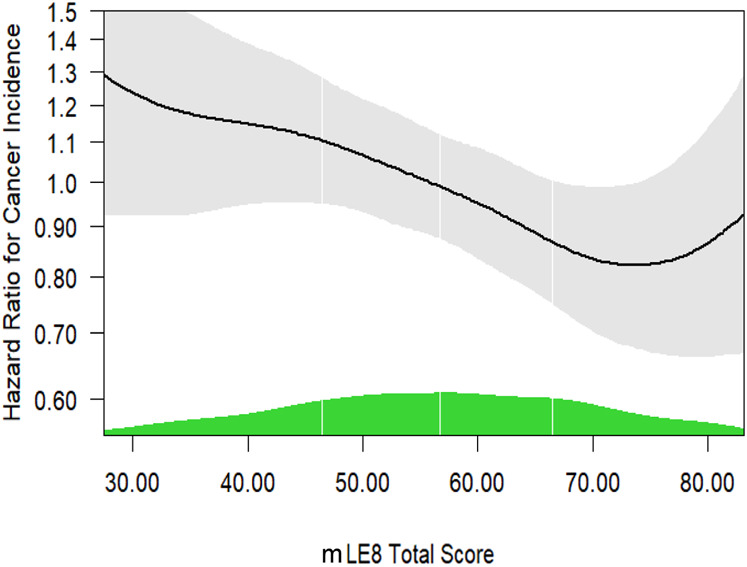
Spline-based hazard ratios for incident cancer associated with modified Life's Essential 8 (mLE8). The curve is based on a spline-based Cox model, adjusted for age, sex, ethnicity, pre-existing CVD, education, and neighborhood socioeconomic status. The grey area represents a 95% confidence interval. The histogram at the bottom shows the relative distribution of mLE8 scores. The *y-axis* is presented on a logarithmic scale. *P* values for the mLE8-cancer incidence association are 0.027 for linearity and 0.344 for nonlinearity.

Using spline methodology, mLE8 was inversely associated with cancer incidence in a linear fashion; as mLE8 increased, cancer risk decreased (p for linearity = 0.027), with no evidence of nonlinearity (Figure 2).

**FIGURE 3:**
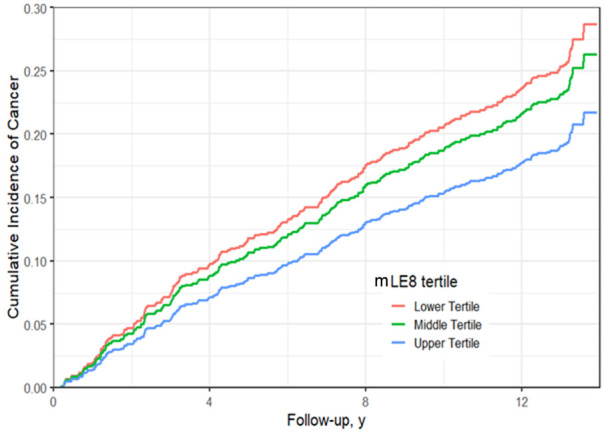
Cumulative incidence function (CIF) of incident cancer across modified Life’s Essential 8 (mLE8) tertiles among CVD-free individuals: covariate-adjusted analysis with death as a competing event. Estimates are adjusted for age, sex, ethnicity, education, and neighborhood socioeconomic status; p for trend = 0.05.

The multivariable adjusted CIF of cancer during follow-up across mLE8 tertiles among CVD-free individuals, with death treated as a competing event, is presented in Figure 3. Cancer incidence increased with decreasing LE8 tertiles (p for trend = 0.05). This pattern indicates that poorer CVH is associated with a higher cumulative risk of cancer in CVD-free individuals.

To evaluate the robustness of our findings, we conducted several sensitivity analyses. In analyses using a modified CVH score that excluded the smoking component, an inverse association persisted, particularly among participants without pre-existing CVD, although it was attenuated (Table 4). In an additional sensitivity analysis, the cholesterol component of the mLE8 was reclassified based on self-reported physician-diagnosed hypercholesterolemia or use of lipid-lowering therapy. After adjustment for age, sex, ethnicity, pre-existing CVD, education, and nSES, the HR (95% CI) for cancer per 1 SD increase in the alternative mLE8 score was 0.86 (0.77–0.97), indicating a slightly stronger inverse association than that observed in the main analysis. In an additional sensitivity analysis, the lipid component was excluded from the LE8 score. After multivariable adjustment, the association between the modified index (excluding the lipid component) and cancer incidence was slightly stronger than in the primary analysis (Table 4). In an analysis restricted to participants with complete data on all LE8 components (n = 1,211), the results were consistent with the primary analysis, although a slight attenuation of the association was observed, with wider confidence intervals reflecting the reduced sample size (Table 4).

## Discussion

### Principal findings

In this nationwide cohort of community-dwelling older adults with a mean age of 75 years, better CVH assessed by an mLE8 was associated with a significantly lower long-term risk of cancer. Over a median follow-up of 11.7 years, each 1 SD increase in mLE8 (about 14 points in our cohort) was associated with a 12% lower risk of cancer in the adjusted model. Because LE8 represents an average of eight component scores, a change of Δ points in one component changes the overall score by Δ/8 points. For example, increasing physical activity from 30-59 to ≥150 min per week increases total LE8 by 7.5 points. Similarly, improving BMI from 35.0-39.9 to 25.0-29.9 kg/m^2^ or shifting diet quality from the 25th-49th to the 75th-94th percentile increases total LE8 by approximately 7 points. Improvements across two such domains would approximate a 1 SD increase and may be associated with a clinically meaningful reduction in the long-term risk of cancer. Importantly, the inverse association was primarily evident among participants free of pre-existing CVD and remained robust in competing risk and sensitivity analyses, with a linear dose-response across the mLE8 distribution.

### Comparison with previous studies

Epidemiological evidence suggests that up to 20% of malignancies could be related to obesity (Koene et al., 2016). Related to this, numerous studies have linked diabetes mellitus to cancer risk and its progression. An umbrella review concluded that there is robust evidence supporting associations between diabetes mellitus and breast, intrahepatic cholangiocarcinoma, colorectal, and endometrial cancers (Tsilidis et al., 2015). Hypertension has also been associated with several specific cancer types, particularly renal cell carcinoma (Grossman et al., 2002). Several studies have demonstrated an inverse association between low-density lipoprotein cholesterol and cancer risk (Koene et al., 2016). Higher diet quality, as assessed using HEI-2015, was associated with lower CVD (Xu et al., 2020) and cancer risk (Julian-Serrano et al., 2022; Schwingshackl et al., 2018). Physical activity has positive effects across all sociodemographic groups in preventing numerous chronic diseases, including CVD (Lavie et al., 2019) and cancer (Cohen et al., 2020a).

Smoking is a preventable and strong risk factor for multiple cancer types. The American Cancer Society estimates that smoking is responsible for 30% of all cancer-related deaths in the United States (Koene et al., 2016). Smoking cessation is the most effective measure for preventing cancer and CVD (Lotan et al., 2017; Messner and Bernhard, 2014). Population-level studies have shown that poor sleep quality and abnormal sleep duration are associated with an increased risk of cancer (Li et al., 2022; Song et al., 2021) and CVD (Lloyd-Jones et al., 2022; Ashri et al., 2024).

### Potential biological mechanisms

Although commonly viewed as two distinct disease entities, CVD and cancer share multiple risk factors (e.g., smoking, obesity, diabetes mellitus, and sedentary lifestyle) and exhibit important biological overlap, supporting the concept of a shared pathophysiology (Koene et al., 2016). Several mechanisms may underlie the development of both conditions, including chronic inflammation, metabolic remodeling, clonal hematopoiesis, dysregulated angiogenesis, and alterations in the extracellular matrix and stromal environment (de Boer et al., 2020). Clonal hematopoiesis has been causally linked to increased risks of cancer and all-cause mortality, while somatic mutations in hematopoietic cells and macrophages may accelerate atherosclerosis and CVD progression. Interleukin-1β and downstream interleukin-6 signaling pathways contribute to tumor-promoting inflammation and have also been implicated in CVD development. Similarly, vascular endothelial growth factor is involved in endothelial dysfunction and angiogenesis and is highly expressed in both malignancy and CVD (Jiang et al., 2024). The complex immune and reparative processes following myocardial infarction, including monocyte activation and altered immune surveillance, may further contribute to a pro-tumorigenic milieu. Heart failure after myocardial infarction has been associated with immune dysregulation, which may impair tumor immune surveillance and promote tumor growth (Hasin et al., 2016). These shared inflammatory and immune pathways provide a plausible biological basis for the observed relationship between overall CVH and subsequent cancer risk.

Notably, in our study, the inverse association between LE8 and cancer incidence was stronger among individuals free of pre-existing CVD than among those with established CVD. Several explanations may account for this finding. First, competing risks may play a role: individuals with established CVD are at higher risk of cardiovascular mortality, which may reduce the probability of surviving long enough to develop or be diagnosed with cancer, thereby attenuating the observed association (Huether et al., 2025). Second, survivorship bias may contribute, as individuals with prevalent CVD who are included in long-term observational cohorts may represent a selected subgroup with greater biological resilience or more intensive medical management, potentially influencing observed associations (Saracci, 2007). Third, differences in clinical management and healthcare utilization may also be relevant. Individuals with established CVD are more likely to receive ongoing medical care, including risk factor modification and pharmacologic treatment as part of secondary prevention, which may mitigate the impact of lifestyle-related exposures on subsequent cancer risk (Bahit et al., 2023). Finally, underlying pathophysiological differences may contribute. In individuals with established CVD, processes such as chronic inflammation, oxidative stress, and vascular damage may already be advanced, potentially diminishing the relative influence of current CVH status on cancer development. This is consistent with prior work highlighting shared but complex biological pathways between CVD and cancer (Qiu et al., 2026).

Colorectal cancer, a major digestive malignancy, was the most common site in our cohort. Shared mechanisms linking CVH and digestive cancers may involve oxidative stress, chronic low-grade inflammation, and diet-related alterations in the gut microbiome. Diet quality may influence colorectal carcinogenesis through inflammatory signaling pathways and changes in the intestinal microbial environment. In addition, long non-coding RNAs, which regulate gene expression through epigenetic and transcriptional mechanisms, have been implicated in both CVD and cancer progression, including gastric metastasis (Jiang et al., 2024).

### Strengths and limitations

This population-based cohort study included a nationwide, representative sample of community-dwelling older Israeli adults with a mean age of approximately 75 years, followed for up to 15 years. This age group bears the greatest burden of cancer, CVD, and multimorbidity, yet remains underrepresented in epidemiologic studies of CVH metrics and cancer risk. The dataset was derived from a comprehensive national survey covering a wide range of health, nutritional, clinical, cognitive, and sociodemographic domains, with representation of diverse ethnic and socioeconomic communities in Israel. Linkage to the Israel National Cancer Registry, which has high completeness, enabled accurate and virtually complete long-term ascertainment of incident cancer. We used a modified AHA LE8 composite construct, incorporating eight health behaviors and clinical factors, to capture overall CVH. Given the advanced age of the cohort, analyses accounted for death as a competing event.

Several limitations should be considered. First, causal inference is limited because of the observational design. Second, residual confounding cannot be excluded because we lacked information on several relevant covariates, including family history, alcohol consumption, cancer screening practices, ionizing radiation, occupational and environmental exposures, viral infections, and reproductive factors (Colditz et al., 2012). Regarding alcohol use, the HEI-2015 (used to derive the diet component of LE8) does not include alcohol as a separate component but incorporates energy from alcohol within total energy intake (Goshen et al., 2023). In addition, alcohol consumption in this cohort was relatively low, with only ∼15% of participants reporting intake at least once per week, suggesting that alcohol is unlikely to be a major confounder. Regarding environmental exposures, prior analyses in this cohort have demonstrated a very weak association between air pollution exposure and cancer risk (Cohen et al., 2020b), suggesting that such exposures are unlikely to substantially influence the observed associations. Nevertheless, residual confounding should be considered when interpreting our findings. Third, several LE8 components were adapted to the available MABAT ZAHAV data. Specifically, dietary cholesterol was used as a proxy for the lipid component, and self-reported diabetes was used as a proxy for the glucose component. These substitutions may introduce measurement error and limit comparability with studies using the standard AHA LE8 definitions. However, such misclassification is likely to be largely non-differential with respect to subsequent cancer incidence and therefore would be expected to attenuate the observed associations. In a sensitivity analysis excluding the lipid component, the association with cancer incidence was slightly stronger, suggesting that misclassification in this component may have diluted the association in the primary analysis. Fourth, changes in health behaviors and risk factor profiles during follow-up were not captured. Components of LE8, such as diet, physical activity, and smoking, may vary over time, particularly in older adults, due to changes in health status, functional capacity, and clinical conditions (Goshen et al., 2022; Lotan et al., 2017; Gerber et al., 2025). As our analysis relies on baseline measurements, this may introduce exposure misclassification. However, such misclassification is likely to be non-differential and therefore expected to bias the observed associations toward the null (White et al., 1998). Fifth, the number of events for specific cancer sites was limited, precluding site-specific analyses. Lastly, we allowed estimation of the mLE8 score as long as ≥5 components were available. While this approach maximized sample size, it may introduce heterogeneity in exposure assessment. However, a complete-case analysis yielded consistent results, supporting the robustness of our findings.

### Public health implications

The burden of cancer can be reduced through changes in individual and population-level behaviors, supported by coordinated public health efforts (Colditz et al., 2012). In aging populations, CVD and cancer account for the majority of morbidity, mortality, and healthcare utilization, imposing a substantial economic burden on society and healthcare systems (Colditz et al., 2012; Townsend et al., 2022). Recognizing their shared determinants may enable more effective and integrated prevention strategies, particularly for high-risk older adults. Healthcare professionals caring for older adults should be aware that poor CVH and established CVD may contribute to elevated cancer risk and cancer-related mortality.

While many prior studies have focused on individual risk factors such as body weight, diet, smoking, or alcohol consumption (Townsend et al., 2022; Chlebowski et al., 2020; Lisevick et al., 2021), our study evaluated multiple health behaviors and cardiometabolic factors within an integrated mLE8 framework, while accounting for sociodemographic characteristics. In the context of an aging population, incorporating LE8 into routine clinical and public health assessments may facilitate personalized prevention strategies targeting both CVD and cancer risk. Future studies with larger sample sizes are needed to further clarify the association between LE8 and site-specific cancers in older adults.

## Conclusion

In this nationwide cohort of community-dwelling older adults with a mean age of 75 years, higher LE8 scores were independently associated with lower long-term risk of cancer. Each incremental improvement in CVH translated into a measurable reduction in cancer risk, with the association particularly evident among individuals free of pre-existing CVD. These findings extend the relevance of the AHA LE8 construct beyond cardiovascular outcomes and suggest that maintaining favorable CVH in later life may contribute to cancer prevention. In aging populations, where the burden of CVD, cancer, and multimorbidity is greatest, integrated strategies targeting overall CVH may offer a pragmatic approach to reducing the combined burden of CVD and cancer.

## Data Availability

Survey data (excluding outcome variables) are available upon request: https://www.gov.il/en/pages/mabat-2005-2006-a.
